# Natural senolytic activity of *Rhodiola rosea* extract alleviates age-associated phenotypes via paraptosis

**DOI:** 10.1016/j.isci.2026.115607

**Published:** 2026-04-04

**Authors:** Ryo Furuuchi, Yohko Yoshida, Goro Katsuumi, Takaaki Furihata, Yusuke Joki, Chieh-Lun Hsiao, Masayoshi Suda, Hana Saito, Tamano Kumazaki, Hidefumi Makabe, Manabu Abe, Ippei Shimizu, Tohru Minamino

**Affiliations:** 1 Advanced Research Institutes, Bourbon Corporation, Niigata 956-0841, Japan; 2 Department of Advanced Senotherapeutics, Juntendo University Graduate School of Medicine, Bunkyo-ku, Tokyo 113-8431, Japan; 3 Department of Cardiovascular Biology and Medicine, Juntendo University Graduate School of Medicine, Bunkyo-ku, Tokyo 113-8431, Japan; 4 Department of Cardiovascular Medicine, Niigata University Graduate School of Medical and Dental Sciences, Niigata 951-8510, Japan; 5 Department of Agriculture, Graduate School of Science and Technology, Shinshu University, Kami-ina, Nagano 399-4598, Japan; 6 Department of Animal Model Development, Brain Research Institute, Niigata University, Niigata 951-8510, Japan; 7 Department of Cardiovascular Aging, National Cerebral and Cardiovascular Center Research Institute, Suita, Osaka 564-8565, Japan; 8 Department of Cardiovascular Medicine, National Cerebral and Cardiovascular Center, Suita, Osaka 564-8565, Japan

**Keywords:** Biological sciences, Molecular biology, Pharmacology

## Abstract

The accumulation of senescent cells drives age-related diseases, and their removal (senolysis) has been reported to ameliorate pathological aging phenotypes. Here, we identified *Rhodiola rosea* extract (Rosea) as a senolytic agent through screening of edible natural products. In mice, Rosea eliminated irradiation-induced senescent cells and reduced the burden of senescent cells in adipose tissue during obesity, as well as in adipose tissue, skin, and skeletal muscle during aging. These effects were accompanied by improvements in metabolic abnormalities, physical function, skin abnormalities, and behavioral impairments. We further identified oligomers of epigallocatechin (EGC) and epigallocatechin gallate (EGCG), specifically EGC-EGCG and EGCG-EGCG, as the senolytic components. EGC-EGCG targeted vulnerabilities in calcium dynamics between the endoplasmic reticulum and mitochondria in senescent cells, thereby inducing paraptosis-like cell death. These findings suggest that Rosea, containing EGC-EGCG and EGCG-EGCG, represents a natural senolytic candidate capable of delaying, mitigating, or preventing the progression of age-related pathologies.

## Highlights

• *Rhodiola rosea* has senolytic activity
• *Rhodiola rosea* improves aging-related phenotypes in obese and aged mice
• EGCG oligomers in *Rhodiola rosea* eliminate senescent cells by inducing paraptosis

## Graphical abstract

**Figure:**
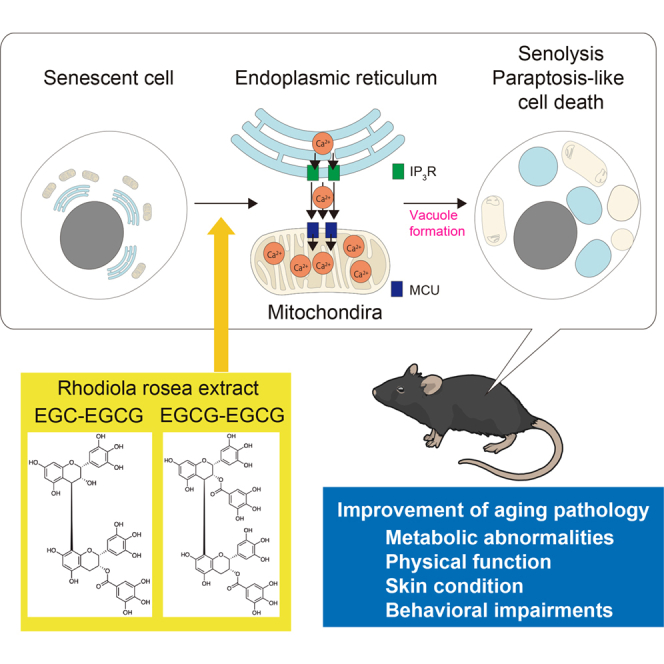

## Introduction

Cellular senescence, a state of stable cell-cycle arrest triggered by various forms of stress, has emerged as a key contributor to aging and age-related diseases, making it an attractive therapeutic target. Senescent cells secrete a range of proinflammatory factors, collectively known as the senescence-associated secretory phenotype (SASP), which can drive chronic tissue inflammation, functional decline, and the progression of age-related pathologies.^1^^,^^2^^,^^3^ In both humans and mice, the accumulation of senescent cells within atherosclerotic lesions has been implicated in vascular inflammation and dysfunction, and inhibition of the p53/p21 pathway has been shown to improve vascular function and attenuate atherosclerosis progression.^4^^,^^5^^,^^6^ Furthermore, in the context of obesity, the accumulation of senescent cells in visceral adipose tissue has been linked to increased inflammation and insulin resistance; notably, inhibition of the p53/p21 pathway in adipose tissue has been reported to attenuate metabolic dysfunction.^7^^,^^8^ Cellular senescence also plays a critical role in tumor suppression, yet paradoxically exhibits pro-tumorigenic properties under certain conditions; therefore, direct inhibition of senescence-regulating factors must be approached with caution.^9^ More recently, it has been suggested that the selective elimination of senescent cells (senolysis) not only ameliorates age-associated pathologies but also extends healthspan without increasing cancer risk.^10^ Consequently, there has been growing interest in developing senolytic agents that selectively target and eliminate senescent cells.^11^^,^^12^^,^^13^

To date, most senolytic agents have been developed on the basis of inhibition of antiapoptotic pathways activated in senescent cells.^14^ However, there is an increasing demand for more accessible and safer compounds. In this study, we screened food-derived materials for senolytic activity and identified *Rhodiola rosea* extract (Rosea), which contains oligomers of epigallocatechin (EGC) and epigallocatechin gallate (EGCG).

## Results

### Rosea identified as a senolytic agent

To identify candidate senolytic compounds, we screened 481 edible plant-derived materials. First, we defined human umbilical vein endothelial cells (HUVECs) with a passage number of 14 or higher as senescent cells. Compared to low-passage cells (passage number <5), these senescent HUVECs exhibited elevated markers of cellular senescence, including increased senescence-associated β-galactosidase (SA-β-gal) activity and higher mRNA expression levels of *CDKN1A* (which encodes p21) and *CDKN2A* (which encodes p16^INK4a^), two canonical cyclin-dependent kinase inhibitor genes that are widely used markers of cellular senescence (Figures S1A and S1B).

**Figure 1:**
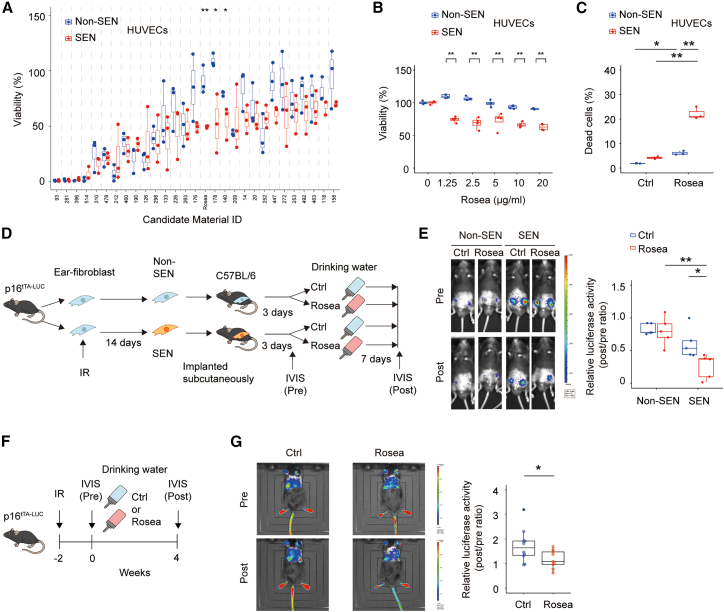
Evaluation of the senolytic activity of Rosea (A) Secondary screen to assess senolytic selectivity in HUVECs. Twenty-nine candidate extracts identified in the primary screen were tested at 12.5 μg/mL in non-senescent (Non-SEN) and senescent (SEN) HUVECs, and cell viability was measured to evaluate senolytic selectivity (*n* = 3 per group). (B) Viability of non-senescent (Non-SEN) and replicative senescent (SEN) human umbilical vein endothelial cells (HUVECs) after treatment with *Rhodiola rosea* extract (Rosea). Cell viability was assessed using the CellTiter-Glo assay (*n* = 4 per group). (C) Flow cytometric analysis of cell death in Non-SEN and SEN HUVECs treated with Rosea, using propidium iodide (PI) staining. The percentage of PI-positive cells was quantified (*n* = 3 per group). (D) Schematic representation of the *in vivo* senescent cell elimination assay using transplanted senescent cells derived from *Cdkn2a*^2A-lox-tTA−2A−Luc^ (p16^tTA−Luc^) mice. Senescence was induced by X-ray irradiation (IR), and cells were transplanted subcutaneously into recipient mice. Mice received Rosea (0.05%) in drinking water for 7 days; control (Ctrl) mice received regular water. Bioluminescence from p16-driven luciferase was measured before and after treatment. (E) Quantification of relative luciferase activity in transplanted p16^tTA−Luc^ cells, expressed as the post-treatment signal divided by the pre-treatment signal (*n* = 5 per group). (F) Schematic representation of the *in vivo* senescence induction protocol using p16^tTA−Luc^ mice. Mice were subjected to X-ray irradiation (IR) to induce cellular senescence, and luciferase activity driven by the p16 promoter was measured to evaluate senescent cell burden. (G) Relative luciferase activity was calculated as the ratio of post-treatment to pre-treatment signals in the IR-induced senescent p16^tTA−Luc^ mice (*n* = 10 for Ctrl, *n* = 9 for Rosea). Statistical analysis was performed using two-tailed Student’s *t* test (A, B, and G) or two-way ANOVA followed by Tukey’s multiple comparisons test (C and E). Sample sizes (n) indicate independent biological replicates for *in vitro* experiments and mice for *in vivo* experiments. P∗ <0.05; P∗∗< 0.01. Data are presented as box-and-whisker plots indicating the full range (whiskers), interquartile range (box), median (solid line), and individual data points (dots).

We conducted a primary screen of plant-derived extracts using senescent HUVECs as a cellular model. In total, 481 extracts were evaluated at a single concentration of 12.5 μg/mL. This concentration was selected as a practical primary screening dose to enable direct comparison across the entire library while maintaining the final DMSO concentration within a non-toxic and consistent range. Following treatment, cell viability in senescent HUVECs was quantitatively assessed (Figure S1C). Based on the results of the primary screen, 29 extracts were prioritized for secondary evaluation using a predefined operational cutoff of ≤50% viability in senescent HUVECs relative to the DMSO-treated control. To assess senolytic selectivity, these 29 candidate extracts were subsequently tested in parallel in non-senescent and senescent HUVECs (Figure 1A). Several candidates exhibited preferential senolytic activity in this secondary screen. Considering practical factors such as availability and feasibility for follow-up studies, including reproducible supply and quality control, we selected *Rhodiola rosea* extract (Rosea) for subsequent experiments. Consistent with this selection, Rosea preferentially reduced the viability of senescent cells compared with non-senescent controls (Figures 1B and 1C). In senescent HUVECs, Rosea treatment did not significantly alter the expression levels of CDKN1A or CDKN2A compared with control-treated cells (Figure S1D), suggesting that Rosea does not reverse the senescent phenotype under these conditions.

To evaluate whether Rosea could eliminate senescent cells *in vivo*, we utilized a senescence-tracking model. We generated a *Cdkn2a* (p16^Ink4a^) knock-in reporter mouse in which a 2A-lox-pA-Neo-lox-tTA-2A-luciferase (Luc) cassette was inserted into the endogenous *Cdkn2a* locus. Upon Cre-mediated recombination, the STOP/*Neo* cassette is excised, yielding the Cre-excised allele (p16^tTA−Luc^; *Cdkn2a*^2A-lox-tTA−2A−Luc^), in which tTA and firefly luciferase are expressed under the control of the endogenous *Cdkn2a* (p16^Ink4a^) promoter (Figure S1E). Cellular senescence was induced *ex vivo* in ear-derived fibroblasts isolated from p16^tTA−Luc^ mice by X-ray irradiation, and the establishment of a senescent phenotype was confirmed by increased expression of senescence markers and enhanced p16-driven reporter activity (Figures S1F and S1G). We then assessed the direct effects of Rosea on irradiated (senescent) versus non-irradiated (non-senescent) fibroblasts *in vitro*. Following irradiation, cells were treated with Rosea for 48 h, after which cell viability was quantified. Under these conditions, Rosea preferentially reduced the viability of senescent fibroblasts, consistent with senolytic activity (Figure S1H). For the *in vivo* assay, to determine whether the senolytic activity observed *in vitro* could be recapitulated *in vivo*, senescent and non-senescent fibroblasts were embedded in Matrigel and transplanted subcutaneously into wild-type C57BL/6 mice (Figure 1D). Robust luciferase signals were detected from the transplanted senescent cells. Importantly, in mice treated with Rosea, these luciferase signals were significantly reduced, suggesting *in vivo* elimination of the senescent cells (Figure 1E).

To further assess whether senescent cells induced *in vivo* could also be eliminated, we irradiated the skin of p16^tTA−Luc^ mice with 14 Gy of X-rays to induce cellular senescence (Figure S1I), and administered Rosea 2 weeks later (Figure 1F). Irradiation resulted in increased luciferase activity, although this increase was significantly suppressed in Rosea-treated mice, suggesting a reduction in the p16-positive senescent cell population (Figure 1G). Together, these results suggests that Rosea exhibits senolytic activity both *in vitro* and *in vivo*.

### Metabolic benefits of Rosea in a HFD-induced obesity model

**Figure 2:**
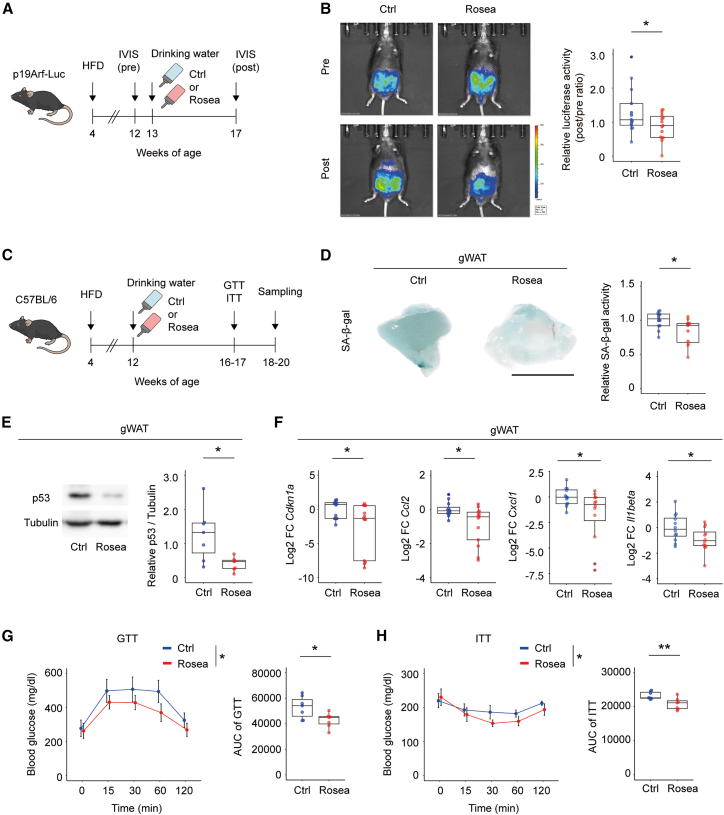
Effects of Rosea on HFD-induced senescent cells in visceral adipose tissue (A) Scheme for the experimental design using high-fat diet (HFD)-induced obese p19Arf-DTR-luciferase (p19Arf-Luc) mice to evaluate the effect of Rosea on *in vivo* senescent cell elimination. (B) Relative luciferase activity in p19Arf-Luc mice was calculated as the ratio of post-treatment to pre-treatment values, following 4-week administration of Rosea or control (Ctrl) drinking water (*n* = 13, 15). (C) Experimental scheme for evaluating senescence and systemic metabolism using HFD-induced obese C57BL/6 mice treated with Rosea or Ctrl water. (D) Quantification of senescence-associated β-galactosidase (SA-β-gal) activity in gonadal white adipose tissue (gWAT) from the Ctrl and Rosea groups. Representative images are shown. Scale bars, 1 cm (*n* = 12, 11). (E) Relative protein expression levels of p53 in gWAT, as determined by immunoblot analysis and normalized to α-Tubulin (*n* = 7, 7). The unmodified blot image corresponding to Figure 2E is provided in Data S1. (F) Relative mRNA expression levels of senescence (*Cdkn1a*) and SASP (*Ccl2, Cxcl1*, and *Il1β*) markers in gWAT from the Ctrl and Rosea groups (*n* = 12, 12). (G) Glucose tolerance test (GTT) results in the Rosea- and Ctrl-treated mice (*n* = 7, 7). (H) Insulin tolerance test (ITT) results in the Rosea- and Ctrl-treated mice (*n* = 7, 7). Statistical analysis was performed using a two-tailed Student’s *t* test for (B and D−H) and repeated measures ANOVA for (G and H). P∗ <0.05. Sample sizes (n) indicate mice per group. The data are shown as box-and-whisker plots indicating the full range (whiskers), interquartile range (boxes), and median (solid line), with individual data points represented as dots or as mean ± 2 × standard error of the mean (SEM), where appropriate.

To evaluate the effects of Rosea in a physiologically relevant model of senescent cell accumulation associated with lifestyle-related factors, we employed a diet-induced obesity model. A transgenic mouse model (p19^Arf^-Luc) was generated using a phage artificial chromosome containing the mouse *Ink4a/Arf* (*Cdkn2a*) locus. In this construct, the ARF-specific exon 1β was replaced with a cassette encoding the diphtheria toxin receptor (human HB-EGF I117V/L148V) linked to firefly luciferase via a self-cleaving 2A peptide (DTR-2A-Luc).^15^ Mice were then fed a high-fat diet (HFD) for 8 weeks to induce obesity, followed by 4 weeks of Rosea administration via drinking water (Figure 2A). Compared to control mice, Rosea-treated mice exhibited a significant reduction in luciferase signal, suggesting an attenuation of p19^Arf^-associated reporter activity (Figure 2B).

We further validated these effects by using wild-type C57BL/6 mice subjected to HFD feeding (Figure 2C). Rosea administration led to a decrease in SA-β-gal activity in gonadal white adipose tissue (gWAT) (Figure 2D), a reduction in p53 protein expression (Figure 2E), and suppressed expression of *Cdkn1a*, *Ccl2, Il1β*, and *Cxcl1* mRNA (Figure 2F). To evaluate the effect of Rosea on adipose tissue inflammation, we performed a quantitative analysis of crown-like structures (CLS) in gWAT. CLS were significantly reduced following Rosea treatment (Figure S2A). Glucose tolerance and insulin sensitivity tests demonstrated improved glucose metabolism in Rosea-treated mice compared to controls (Figures 2G and 2H). Notably, no significant differences were observed in body weight, gWAT mass, water intake, or food consumption between the groups (Figures S2B–S2E). These findings suggest that Rosea improves HFD-induced metabolic dysfunction and adipose tissue inflammation and is associated with reduced senescence-related readouts in adipose tissue.

### Effects of Rosea in middle-aged and aged mice

**Figure 3:**
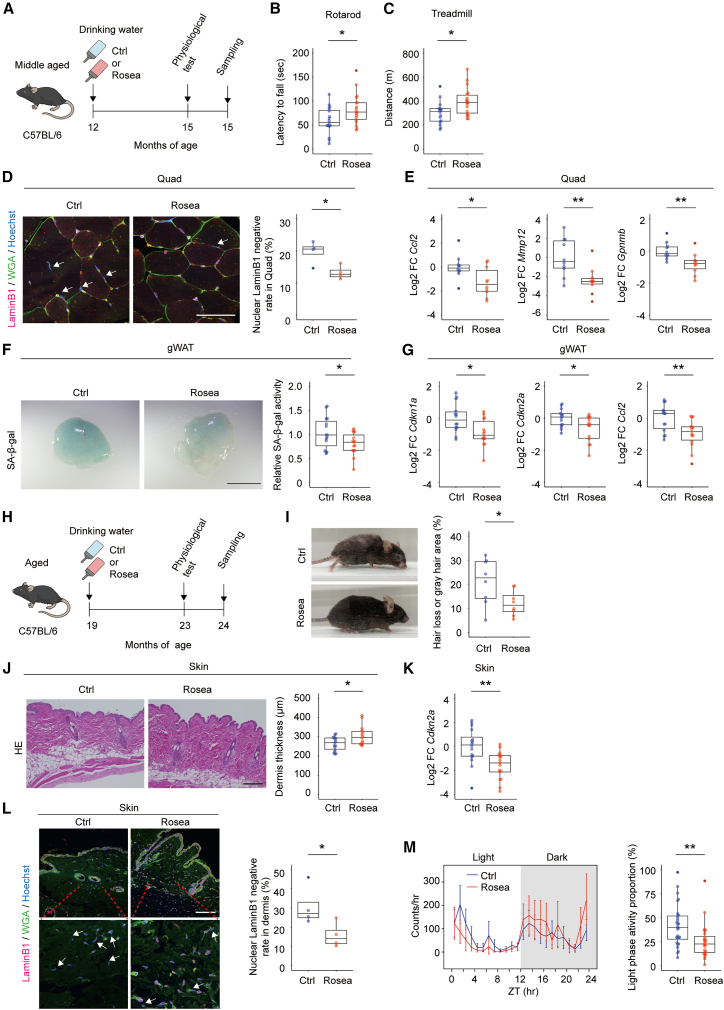
Effects of Rosea in aged and middle-aged mice (A) Schematic illustration of the experimental design to assess the effects of chronic Rosea administration in middle-aged mice. (B) Results of the rotarod test in middle-aged mice treated with either control (Ctrl) or Rosea water. Latency to fall was recorded and averaged across three trials (*n* = 16, 18). (C) Results of the treadmill endurance test in the same cohort. Running distance prior to exhaustion was measured and compared (*n* = 16, 18). (D) Representative immunofluorescence images of LaminB1 in quadriceps muscle (Quad) sections. The proportion of LaminB1-negative nuclei was quantified in the Ctrl and Rosea groups (*n* = 4, 4). Arrows indicate nuclei negative for Lamin B. Scale bars, 50 μm. (E) Relative mRNA expression levels of senescence- and inflammation-related genes *Ccl2*, *Mmp12*, and *Gpnmb* in Quad tissues (*n* = 10, 10). (F) SA-β-gal activity in gonadal white adipose tissue (gWAT) of middle-aged mice after Rosea or Ctrl water administration. Representative whole tissue images are shown. Scale bars, 1 cm (*n* = 15, 15). (G) Relative mRNA expression levels of *Cdkn1a*, *Cdkn2a*, and *Ccl2* in gWAT (*n* = 14, 14). (H) Schematic illustration of the experimental protocol used to evaluate Rosea in aged mice. (I) Representative images of aged mice treated with Ctrl or Rosea water. The percentages of total body surface area affected by hair loss or graying were quantified (*n* = 8, 8). (J) Hematoxylin and eosin (H&E) staining of dorsal skin from aged mice. Dermis thickness was measured and compared between groups (*n* = 15, 14). Scale bars, 100 μm. (K) Relative mRNA expression levels of *Cdkn2a* in skin samples (*n* = 16, 17). (L) Representative immunostaining images of LaminB1 in dorsal skin sections of aged mice. The frequency of LaminB1-negative nuclei was quantified (*n* = 4, 4). Arrows indicate nuclei negative for Lamin B. Scale bars, 100 μm. (M) Left: average hourly activity levels in aged mice measured using a wheel running apparatus. Right: comparison of light-phase (ZT 0–12 h) activity percentages between the Ctrl and Rosea groups (*n* = 28, 24). Statistical analysis was performed using a two-tailed Student’s *t* test. Sample sizes (n) indicate mice per group. P∗ <0.05; P∗∗< 0.01. All data are shown as box-and-whisker plots, with whiskers representing the full range, boxes indicating the 25th–75th percentiles, medians shown as solid lines, and individual data points represented as dots or mean ± 2 × SEM.

To investigate the effects of Rosea on age-associated senescence, we utilized middle-aged C57BL/6 mice in which senescent cells accumulate spontaneously with advancing age. Twelve-month-old mice received Rosea administered *ad libitum* in the drinking water for 12 weeks (Figure 3A). Motor function was evaluated at the end of the treatment period using rotarod (Figure 3B) and treadmill tests (Figure 3C). Mice were sacrificed 2 weeks after completion of the motor assessments for tissue collection and subsequent analyses. Rosea-treated mice exhibited significantly improved motor performance compared to controls. Age-related pathological changes in skeletal muscle were also ameliorated. Loss of nuclear Lamin B1, a hallmark of cellular senescence,^16^ was ameliorated in the Rosea-treated group (Figure 3D). Masson’s trichrome staining revealed a significant reduction in muscle fibrosis (Figure S3A). Moreover, the expression of SASP factors such as *Ccl2* and *Mmp12,* as well as the senescence marker *Gpnmb*,^13^^,^^17^ was significantly downregulated in skeletal muscle following Rosea treatment (Figure 3E). Similar improvements were observed in adipose tissue. SA-β-gal activity (Figure 3F), as well as mRNA expression levels of *Cdkn1a*, *Cdkn2a*, *Ccl2*, and *Gpnmb* (Figure 3G and S3B), and p53 protein levels (Figure S3C), were all significantly reduced. There were no significant differences in body weight, food intake, or other metabolic parameters between groups (Figures S3D–S3H).

To assess the effects of Rosea in aged animals, 19-month-old mice were administered Rosea *ad libitum* in the drinking water for 16 weeks (Figure 3H). Behavioral assessments were conducted at the end of the treatment period, and mice were sacrificed 4 weeks thereafter for downstream analyses. Compared to controls, Rosea-treated mice showed markedly reduced signs of aging, including decreased hair loss and/or graying (Figure 3I). Dermal thickness, a parameter linked to skin collagen content and elasticity,^18^ was significantly increased in the Rosea group, suggesting an improvement in skin quality (Figure 3J). In addition, *Cdkn2a* expression and Lamin B1 loss in the skin^16^^,^^19^ were significantly reduced (Figures 3K and 3L). To evaluate whether Rosea affected age-associated alterations in behavioral patterns, we performed a wheel running test. Aged mice were acclimated for 1 day, after which the number of wheel rotations was recorded hourly. It is known that aged mice exhibit disrupted circadian activity rhythms, characterized by increased activity during the light phase (normally the rest period) and reduced activity during the dark phase (active period).^20^ Although Rosea treatment did not increase the total number of rotations (Figure S3I), it improved the distribution of activity between the light and dark phases (Figure 3M). Furthermore, Rosea administration did not affect body weight, food intake, or water consumption in aged mice (Figures S3J–S3L). These findings further support the notion that Rosea may be beneficial in ameliorating age-related physiological and behavioral decline.

### Identification of EGC-EGCG and EGCG-EGCG as senolytic components in Rosea

**Figure 4:**
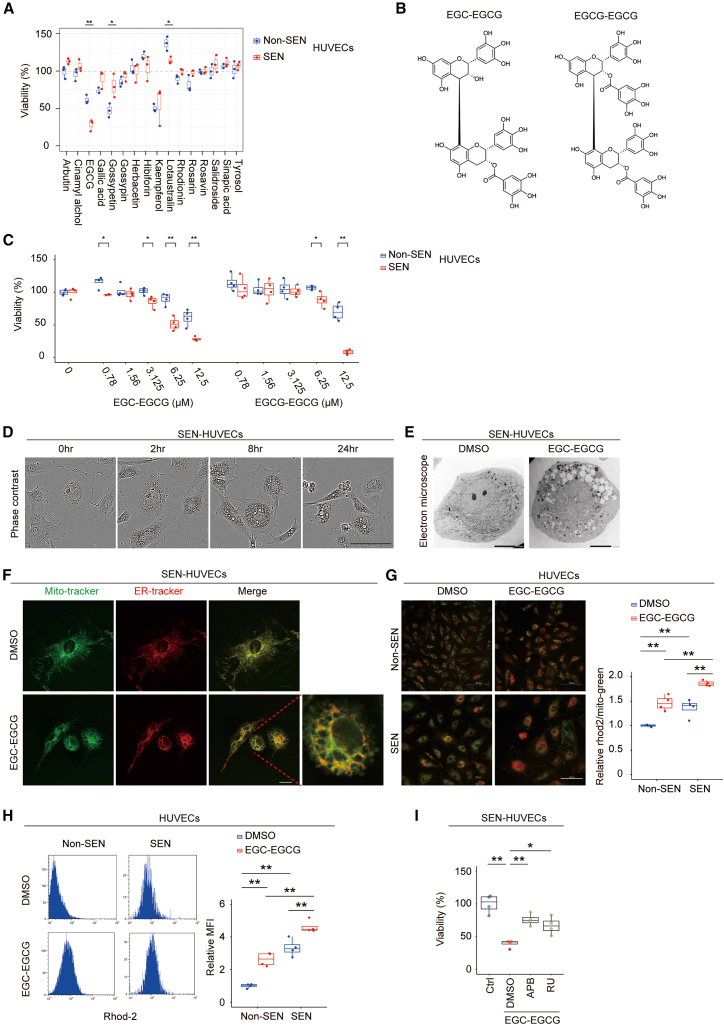
Identification of active components in Rosea and mechanistic analysis of senolytic activity (A) Viability assays of non-senescent (Non-SEN) and replicative senescent (SEN) HUVECs treated with representative compounds found in *Rhodiola rosea* extract (Rosea) at a concentration of 25 μM. Cell viability was assessed after 48 h (*n* = 3 each). (B) Chemical structures of synthesized EGCG oligomers: EGC-EGCG and EGCG-EGCG. (C) Viability assays of Non-SEN and SEN HUVECs treated with EGC-EGCG or EGCG-EGCG (12.5 and 25 μM, 48 h) (*n* = 4 each). (D) Phase-contrast microscopy images showing the time-dependent morphological changes in SEN HUVECs following treatment with EGC-EGCG (12.5 μM). Scale bars, 100 μm. (E) Transmission electron microscopy (TEM) images of SEN HUVECs treated with DMSO or EGC-EGCG (12.5 μM) for 24 h. Scale bars, 10 μm. (F) Confocal images of SEN HUVECs stained with ER-Tracker (red) and MitoTracker Green (green) after treatment with EGC-EGCG (12.5 μM) or DMSO for 24 h. Scale bars, 20 μm. (G) Confocal microscopy images of Non-SEN and SEN HUVECs stained with Rhod-2 AM (red) and MitoTracker Green (green) after 1-h treatment with EGC-EGCG or DMSO. The Rhod-2 signal was normalized to MitoTracker Green, and the ratio was quantified (*n* = 4 each). Scale bars, 100 μm. (H) Flow cytometric analysis of intracellular Ca^2+^ levels using Rhod-2 AM staining after 24-h treatment with EGC-EGCG or DMSO in Non-SEN and SEN HUVECs. Mean fluorescence intensity (MFI) values are shown (*n* = 4 each). (I) Viability of SEN HUVECs pretreated with 2-APB (10 μM), ruthenium red (RU, 2.5 μM), or DMSO (Ctrl) for 1 h, followed by EGC-EGCG (12.5 μM) or DMSO (Ctrl) for 48 h (*n* = 4 each). Statistical significance was determined using the two-tailed Student’s *t* test (A and C) or two-way ANOVA followed by Tukey’s multiple comparisons test (G−I). Sample sizes (n) indicate independent biological replicates per group. P∗ <0.05; P∗∗< 0.01. All data are presented as box-and-whisker plots showing the full data range (whiskers), interquartile range (box), and median (solid line); individual data points are shown as dots.

To identify the active constituents of Rosea, we evaluated its major components, as previously reported.^21^ These compounds were obtained as commercially available purified standards and were individually tested using the same HUVEC viability assay. Among the major components examined, only EGCG selectively reduced the viability of senescent cells (Figure 4A). Previous studies have reported the presence of EGCG- and EGC-related oligomers in Rosea.^21^ We chemically synthesized two such oligomers, EGC-EGCG and EGCG-EGCG (Figure 4B), and evaluated their effects on senescent cells. Both EGC-EGCG and EGCG-EGCG induced cell death in senescent HUVECs and senescent human preadipocytes at lower concentrations than the EGCG monomer (Figures 4C, S4A, and S4B). EGC-EGCG induced senescent cell death in a time-dependent manner (Figure S4C), and similar effects were observed in human preadipocytes rendered senescent by ionizing radiation (Figure S4D–S4F).

Senescent cells treated with EGC-EGCG exhibited a time-dependent increase in characteristic cytoplasmic vacuole formation, which was readily observed by light microscopy (Figure 4D) and further confirmed by transmission electron microscopy (Figure 4E). Similarly, treatment of senescent cells with Rosea also promoted time-dependent vacuolization (Figure S4G). These morphological changes are distinct from classical apoptosis and are instead consistent with a form of non-apoptotic cell death known as paraptosis.^22^^,^^23^ To determine whether the observed cell death was distinct from apoptosis, we examined the effect of the pan-caspase inhibitor Z-VAD-FMK. Pre-treatment with Z-VAD-FMK did not suppress EGC-EGCG-induced cell death in senescent cells, indicating that the cell death mechanism is not attributable to canonical apoptosis (Figure S4H). Paraptosis is thought to result from osmotic imbalance in intracellular organelles, primarily triggered by abnormal calcium dynamics between the endoplasmic reticulum (ER) and mitochondria. Using ER-Tracker and Mito-Tracker, we observed pronounced vacuolization in both the ER and mitochondria of senescent cells treated with EGC-EGCG (Figure 4F). It is well established that paraptosis is associated with rapid mitochondrial calcium accumulation.^24^ Previous studies have shown that senescent cells exhibit impaired coordination of calcium dynamics between the ER and mitochondria.^25^ To assess this, we measured mitochondrial calcium levels using Rhod-2 AM and found that senescent cells exhibited elevated baseline mitochondrial calcium compared to non-senescent cells (Figure 4G). Stimulation with EGC-EGCG significantly increased these calcium levels (Figures 4G and 4H). To evaluate whether the calcium dynamics altered by EGC-EGCG contribute to the senolytic activity of this compound, we utilized 2-aminoethoxydiphenyl borate (2-APB), an inhibitor of the inositol 1,4,5-trisphosphate receptor (IP_3_R), a calcium channel in the ER, and ruthenium red, an inhibitor of the mitochondrial calcium uniporter (MCU). Pre-treatment of senescent cells with 10 μM 2-APB or 2.5 μM ruthenium red significantly suppressed EGC-EGCG-induced cell death (Figure 4I). These findings suggest that EGC-EGCG induces senescent cell death via a paraptosis-like mechanism mediated by dysregulated calcium dynamics between the ER and mitochondria.

## Discussion

The accumulation of senescent cells in tissues is accelerated by DNA damage caused by aging and metabolic stress. This accumulation of senescent cells contributes to the progression of age-related diseases such as diabetes and atherosclerosis. Thus, methods for eliminating senescent cells are being actively studied as both preventive and therapeutic approaches. However, some existing senolytic drugs have been reported to cause adverse effects, and there is a growing need to identify compounds with more favorable toxicity profiles. In this context, we identified Rosea, which contains EGC-EGCG and EGCG-EGCG, as a senolytic agent derived from an edible material with an already established safety profile.^26^

Rosea, a plant known for its adaptogenic properties, has attracted considerable attention for its potential anti-aging effects due to its potent antioxidant activity and stress-reducing capabilities.^27^ Its physiological activities have been attributed to a group of compounds such as salidroside and rosavins (including rosavin and rosarin).^28^ In the present study, however, these conventionally studied components did not exhibit senolytic activity; instead, the observed senolytic activity was limited to EGCG oligomers. The existence of EGCG oligomers in natural products has been reported in only a limited number of studies,^29^^,^^30^ and it is possible that these oligomers are characteristic components of Rosea.

In cancer research, the use of paraptosis inducers has attracted attention as a way to induce cell death in cancer cells that are resistant to apoptosis.^22^^,^^23^ EGC-EGCG, identified in this study, has a mechanism of action distinct from that of conventional senolytics that target anti-apoptotic pathways. Specifically, EGC-EGCG acts on the calcium dynamics between the ER and mitochondria, which become vulnerable during cellular senescence, and induces paraptosis-like cell death. To the best of our knowledge, there have been no previous reports of senolytic agents that induce paraptosis-like cell death, making this an important finding of the present study. Dysfunction of the ER and mitochondria in senescent cells is well known and is being considered as a target for senolysis.^25^^,^^31^ Our findings suggest that paraptosis-like senolysis may be a viable alternative to apoptosis-dependent mechanisms.

This study presents a pioneering approach to extending healthspan through the use of naturally derived senolytics, based on both *in vitro* and *in vivo* data. The potential anti-aging effects of senolysis induced by Rosea and its EGCG oligomers suggest that further translational research on this compound could contribute to a method for achieving healthier and longer life.

### Limitations of the study

The p16-driven reporter activity in our p16tTA-Luc reporter mice does not provide a direct or absolute quantification of senescent cell numbers. We cannot distinguish whether Rosea primarily reduces senescent non-immune cells, modulates macrophage abundance or activation, or affects both cell populations. We were unable to evaluate the *in vivo* effects of EGCG oligomers alone and instead limited our analysis to the effects of Rosea as a whole. Further investigation is required to determine whether EGCG oligomers exert senolytic effects *in vivo*, and whether synergistic interactions with salidroside and rosavins may contribute to the observed efficacy. Because paraptosis lacks universally accepted, specific molecular biomarkers, we acknowledge as a limitation that the mode of cell death cannot be conclusively determined in this study. In addition, all *in vivo* experiments were performed exclusively in male mice, and thus potential sex-dependent differences in the effects of Rosea were not assessed.

## Resource availability

### Lead contact

Further information and requests for resources and reagents should be directed to the lead contact, Tohru Minamino (t.minamino@juntendo.ac.jp).

### Materials availability

This study did not generate new unique reagents.

### Data and code availability

• The data reported in this paper will be shared by the lead contact upon reasonable request.
• This paper does not report original code. No previously unreported custom computer code or mathematical algorithm was used to generate results central to the conclusions.
• Any additional information required to reanalyze the data reported in this paper is available from the lead contact upon reasonable request.

## Acknowledgments

This work was supported by: a Grant-in-Aid for Scientific Research (S) (23H05487) and Grant-in-Aid for Challenging Research (Pioneering) (25K21776) from the Ministry of Education, Culture, Sports, Science and Technology (MEXT); the Japan Agency for Medical Research and Development (AMED) under grant no. JP20ek0210114, and AMED-CREST under grant no. JP20gm1110012; the Moonshot Research and Development Program (21zf0127003s0201, 25zf0127012s1002); AMED FORCE (25gm4010031); the Japan Society for the Promotion of Science A3 Foresight Program (JPJSA3F20230001); the MEXT-Supported Program for the Strategic Research Foundation at Private Universities Japan, Private University Research Branding Project, and Leading Initiative for Excellent Young Researchers; grants from the Science Research Promotion Fund, the 10.13039/501100008664Ono Medical Research Foundation, the 10.13039/501100006680Takeda Medical Research Foundation, and the Terumo Life Science Foundation (to T.M.); and a Grant-in-Aid for Scientific Research (C) (22K08139) (to R.F).

## Author contributions

T.M. contributed to establishing the research concept and research design, wrote the manuscript, and supervised all experiments. R.F. wrote the manuscript, contributed to designing the experiments, and performed most of the experiments. Y.Y., G.K., T.F., Y.J., C.-L.H., and M.S. contributed to the *in vivo* research. H.M., H.S., and T.K. contributed to the synthesis of EGCG oligomers. M.A. contributed to the analyses of the genetic mouse models. I.S. contributed to establishing the research concept and research design.

## Declaration of interests

R.F. is affiliated with Bourbon Corporation as a researcher. This study was conducted with joint research funds provided by Bourbon Corporation. Bourbon Corporation and Juntendo University established the “Department of Advanced Senotherapeutics” as an endowed collaborative research course to conduct this study. T.M. and Y.Y. concurrently serve as members of this department. R.F. and T.M. are inventors on a patent application related to this work.

## Declaration of generative AI and AI-assisted technologies in the writing process

During the preparation of this work, the authors used ChatGPT (OpenAI) in order to improve the clarity and readability of the text. After using this tool, the authors reviewed and edited the content as needed and take full responsibility for the content of the publication.

## STAR★Methods

### Key resources table

**Table:** 

REAGENT or RESOURCE	SOURCE	IDENTIFIER
**Antibodies**		

Anti-p53	Leica, Germany	Cat# NCL-L-p53-CM5p; RRID:AB_2895247
Anti-αTubulin	Cell Signaling, USA	Cat# 2125; RRID:AB_2619646
Horseradish peroxidase (HRP)-conjugated anti-rabbit IgG secondary antibody	Jackson Immunoresearch, USA	Cat# 115-035-144; RRID:AB_2307391
Wheat Germ Agglutinin (WGA) Alxa flour 488	ThermoFisher SCIENTIFIC, USA	Cat# W11261; RRID:N/A
Anti-Lamin B1 [EPR8985(B)]	Abcam, UK	Cat# ab133741; RRID:AB_2616597
Goat Anti-Rabbit IgG H&L Alexa Fluor 647	Abcam, UK	Cat# ab150079; RRID:AB_2722623

**Chemicals, peptides, and recombinant proteins**		

Collagenase type II	Worthington, USA	Cat# CLS2
DMEM	Sigma-Aldrich, USA	Cat# D6046
Penicillin-streptomycin	Gibco, USA	Cat# 15140122
Arbutin	Tokyo Chemical Industry, Japan	Cat# A0522
Cinnamyl Alcohol	Tokyo Chemical Industry, Japan	Cat# C0362
(−)-Epigallocatechin gallate	AdipoGen, Switzerland	Cat# AG-CN2-0063-M025
Gallic acid	Tokyo Chemical Industry, Japan	Cat# G0011
Gossypetin	EXTRASYNTHESE S.A., France	Cat# 1176
Gossypin	Sigma-Aldrich, USA	Cat# SML0761
Herbacetin	Sigma-Aldrich, USA	Cat# SMB00355
Hibifolin	Sigma-Aldrich, USA	Cat# PHL83580
Kaempferol Hydrate	Tokyo Chemical Industry, Japan	Cat# K0018
Lotaustralin	Santa Cruz Biotechnology, USA	Cat# sc-207835
Rhodionin	MedChemExpress, USA	Cat# HY-N0241
Rosarin	Sigma-Aldrich, USA	Cat# SMB00315
Rosavin	Selleck Chemicals, USA	Cat# S3844
Salidroside	MedChemExpress, USA	Cat# HY-N0109
Sinapic acid	Sigma-Aldrich, USA	Cat# D7927
Tyrosol	Sigma-Aldrich, USA	Cat# 188255
Z-VAD-FMK	LKT Laboratories, USA	Cat# Z8401
2-APB	FUJIFILM Wako, Japan	Cat# 013-24911
Ruthenium red	FUJIFILM Wako, Japan	Cat# 189-03701
D-Luciferin	Promega, USA	Cat# P1043
Matrigel	Corning, USA	Cat# 35237
Protease inhibitor cocktail	Roche, Switzerland	Cat# 11873580001
Amersham ECL Prime detection reagent	Cytiva, USA	Cat# RPN2232
Propidium Iodid	BD Biosciences, USA	Cat# 51-66121E

**Critical commercial assays**		

CellTiter-Glo® Luminescent Cell Viability Assay	Promega, USA	Cat# G7571
QIAzol Lysis Reagent	QIAGEN, Netherlands	Cat# 79306
Maxwell® RSC simplyRNA Tissue Kit	Promega, USA	Cat# AS1340
SYBR™ Green Master Mix	Applied Biosystems, USA	Cat# A25742

**Experimental models: Cell lines**		

Mouse ear-fibroblast	This paper	
Human umbilical vein endothelial cell (HUVEC)	Lonza	Cat# cc-2517
Human visceral preadipocytes (preadipocyte)	Lonza	Cat# PT-5005

**Experimental models: Organisms/strains**		

C57BL/6 mice	SLC Japan	N/A
Aged C57BL/6 mice	Provided by the Foundation for Biomedical Research and Innovation at Kobe through the National BioResource Project (NBRP) of the Ministry of Education, Culture, Sports, Science and Technology (MEXT), Japan.	N/A
p19Arf-DTR-luciferase mice (C57BL/6 background)	Obtained from Sugimoto M.	N/A
p16-tTA-luciferase mouse (C57BL/6 background)	Obtained from Abe M and their colleague.	N/A

**Oligonucleotides**		

*RPLP0*: 5′-TCTACAACCC TGAAGTGCTTGAT-3′, 5′-CAATCTGCAGACAGACACTGG-3′	This paper	N/A
*GAPDH*: 5′-CCCCGGTTTCTATAAATTGAGC-3′, 5′-CACCTTCCCCATGGTGTCT-3′	This paper	N/A
*CDKN1A*: 5′-CGAAGTCAG TTCCTTGTGGAG-3′, 5′-CATGGGTTCTGACGGACAT-3′	This paper	N/A
*CDKN2A*: 5′-GTGGACCTGGCTGAGGAG-3′, 5′-CTTTCAATCGGGGATGTCTG-3′	This paper	N/A
*Rplp0*: 5′-GATGCCCATGGGAAGACAG-3′, 5′-ACAATGAAGCATTTTGGATAA-3′	This paper	N/A
*Gapdh*: 5′-AGCTTGTCATCAACGGGAAG-3′, 5′-TTTGATGT TAGTGGGGTC TCG-3′	This paper	N/A
*Cdkn1a*: 5′-TCCACAGCGATATCCAGACA-3′, 5′-GGACATCACCAGGATTGGAC-3′	This paper	N/A
*Cdkn2a*: 5′-CGACGGGCATAGCTTCAG-3′, 5′-GCTCTGCTCTTGGGATTGG-3′	This paper	N/A
*Gpnmb*: 5′-ACGGCAGGTGGAAGGACT-3′, 5′-CGGTGAGTCACTGGTCAGG-3′	This paper	N/A
*Ccl2*: 5′- CATCCACGTGTTGGCTCA -3′, 5′- GATCATCTTGCTGGTGAATGAGT -3′	This paper	N/A
*Mmp12*: 5′- TTGTGGATAAACACTACTGGAGGT -3′, 5′- AAATCAGCTTGGGGTAAGCA -3′	This paper	N/A
*Cxcl*1: 5′- AGACTCCAGCCACACTCCAA -3′, 5′- TGACAGCGCAGCTCATTG -3′	This paper	N/A
*Il-1beta*: 5′- TGTAATGAAAGACGGCACAC -3′, 5′- TCTTCTTTGGGTATTGCTTGG -3′	This paper	N/A

**Software and algorithms**		

Fiji software (ImageJ version 1.53q)	NIH, USA	N/A
SPSS (version 28.0.1.0)	IBM, USA	N/A
ID7000 software (version 1.1.0.11041)	Sony, Japan	N/A
Living Image software (version 4.5.5)	Perkin Elmer, USA	N/A
Skanlt RE software (version 7.0.2)	ThermoFisher SCIENTIFIC, USA	N/A
Design & Analysis software (version 2.6.0)	ThermoFisher SCIENTIFIC, USA	N/A
Amersham imager 680 software (version 2.0)	Cytivia, USA	N/A
EvolutionCapt edge ×64 (v18.12g)	Vilber, France	N/A
BZ-II viewer (version 2.1.00a)	Keyence, Japan	N/A
NIS-Elements AR (version 5.42.06 (buid 1821))	Nikon, Japan	N/A
Incucyte software (version 2022B rev2)	Sartorius, Germany	N/A
RStudio (version 2024.04.1 + 748)	Posit, USA	N/A
R (version 4.4.0)	R project	N/A

**Other**		

VARIOSKAN LUX multimode reader	ThermoFisher SCIENTIFIC, USA	N/A
Living Image software Version 4.5.5	Perkin Elmer, USA	N/A
Amersham Imager 680	Cytivia, USA	N/A
Fusion Solo S imaging system	Vilber, France	N/A
Spectral Cell Analyzer ID7000	SONY, Japan	N/A
Glucose analyzer	Sanwa Kagaku Kenkyusho, Japan	N/A
Rotarod test	O’Hara & Co., Japan	Cat# RRAC-3002
Running wheel system	Muromachi Kikai, Japan	Cat# MK-713

### Experimental model and study participant details

#### Cell culture

Human umbilical vein endothelial cells (HUVECs) and human visceral preadipocytes were purchased from Lonza and cultured according to the manufacturer’s protocol. These commercially obtained cells were authenticated by the supplier and were certified by the supplier as negative for mycoplasma contamination at the time of purchase. Mouse ear fibroblasts were isolated as described above. Mouse fibroblasts were maintained in DMEM supplemented with 10% FBS and 1% P/S. Senescent HUVECs were defined as cells that had undergone at least 14 passages, and those with fewer than 5 passages were used as young controls. For human preadipocytes and mouse ear fibroblasts, cells with less than 5 passages were categorized as young cells. Senescence was induced by exposing cells to 10 Gy of X-ray irradiation (IR), followed by incubation for 14 days before use.

#### Animal models

All animal experiments were reviewed and approved by the Animal Experiment Committees of Niigata University (approval number: SA00601) and/or Juntendo University (approval number: 1656), and conducted in accordance with protocols authorized by the respective institutional presidents. Most C57BL/6 mice were obtained from SLC Japan. Aged C57BL/6 mice were provided by the Foundation for Biomedical Research and Innovation at Kobe through the National BioResource Project (NBRP) of the Ministry of Education, Culture, Sports, Science and Technology (MEXT), Japan.

Mice were maintained in a specific pathogen-free facility under controlled conditions (temperature: 20°C–26°C; humidity: 40–60%) with a 12-h light/dark cycle. For senescent cell-tracking studies, we used two transgenic reporter models: (1) the *Cdkn2a*^2A-lox-tTA−2A−Luc^ (p16^tTA−Luc^) mouse, in which a tTA-luciferase cassette was knocked in immediately upstream of the endogenous p16 stop codon, and (2) the p19Arf-DTR-luciferase mouse, which expresses both the diphtheria toxin receptor (human HB-EGF I117V/L148V) and luciferase from the *Cdkn2a* locus, kindly provided by Dr. Sugimoto.^15^

Unless otherwise specified, mice were given *ad libitum* access to standard chow diets (CE-2, CLEA Japan; or CRF-1, Oriental Yeast Co., Ltd.) and filtered tap water. For experimental groups, Rhodiola rosea root extract (Rosea; ASK Intercity Co., Ltd.) was dissolved in drinking water at a final concentration of 0.05% (w/v), filtered through a 0.2-μm membrane to remove insoluble particulates, and administered *ad libitum*. Drinking water was replaced twice weekly. Control mice received untreated drinking water under identical housing conditions.

For diet-induced obesity experiments, mice were fed a high-fat diet (HFD32; CLEA Japan) beginning at 4 weeks of age. Details of diet duration and experimental timelines are described in the respective figure legends.

All experiments were conducted using male mice. Anesthesia was administered as needed using either a mixture of medetomidine, midazolam, and butorphanol, or isoflurane. All anesthesia procedures were carried out in accordance with institutional animal welfare guidelines, ensuring minimal discomfort to the animals.

### Method details

#### Plant materials and extract preparation

Plant-derived materials were provided by the National Institutes of Biomedical Innovation, Health and Nutrition (NIBIOHN, Japan), which maintains a curated library of edible plant resources. The library comprises botanically authenticated plant species with documented historical dietary use and established safety profiles, enabling systematic evaluation of bioactive potential in a translational context.

A total of 481 edible plant-derived materials were included in this study. Extracts were prepared from various plant parts, including leaves, stems, flowers, fruits, seeds, roots/rhizomes/tubers/bulbs, bark, branches, and in some cases whole plants. Plant materials were processed and extracted according to standardized protocols established by NIBIOHN to ensure batch consistency and reproducibility. Extracts were dissolved in DMSO at defined stock concentrations and stored under controlled conditions until use.

#### Cell isolation from mouse tissue

Ear skin was excised from 4-week-old p16^tTA−Luc^ mice and sterilized with 70% ethanol. The tissue was then minced and digested with a solution containing 2 mg/mL collagenase type II (Worthington, CLS2) and 1 mM CaCl_2_ in phosphate-buffered saline (PBS) for 20–30 min at 37°C. The resulting cell suspension was filtered through a 40 μm nylon mesh and centrifuged. Cells were resuspended in Dulbecco’s Modified Eagle Medium (DMEM; Sigma, D6046) supplemented with 10% fetal bovine serum (FBS) and 1% penicillin-streptomycin (P/S; Gibco, 15140122), then plated in culture dishes. Proliferating adherent cells were expanded and used as mouse ear-derived fibroblasts.

#### Cell viability assay

Cells were seeded in black 96-well plates and incubated overnight to allow adherence. Following treatment with the indicated compounds, cell viability was assessed using the CellTiter-Glo Luminescent Cell Viability Assay (Promega, G7571) in accordance with the manufacturer’s instructions. Luminescence intensity was measured using a VARIOSKAN LUX multimode reader (Thermo Scientific). The viability of the dimethyl sulfoxide (DMSO)-treated control group was set at 100%, and the relative viability of each treatment group was calculated accordingly.

The following compounds were purchased and evaluated for their effects on cell viability: Arbutin (Tokyo Chemical Industry, A0522), Cinnamyl Alcohol (Tokyo Chemical Industry, C0362), (-)-Epigallocatechin gallate (AdipoGen, AG-CN2-0063-M025), Gallic acid (Tokyo Chemical Industry, G0011), Gossypetin (EXTRASYNTHESE S.A., 1176), Gossypin (Sigma-Aldrich, SML0761), Herbacetin (Sigma-Aldrich, SMB00355), Hibifolin (Sigma-Aldrich, PHL83580), Kaempferol Hydrate (Tokyo Chemical Industry, K0018), Lotaustralin (Santa Cruz Biotechnology, sc-207835), Rhodionin (MedChemExpress, HY-N0241), Rosarin (Sigma-Aldrich, SMB00315), Rosavin (Selleck Chemicals, S3844), Salidroside (MedChemExpress, HY-N0109), Sinapic acid (Sigma-Aldrich, D7927), and Tyrosol (Sigma-Aldrich, 188255). EGC-EGCG and EGCG-EGCG were synthesized based on previously reported methods.^32^^,^^33^

For viability assays involving inhibitors, HUVECs were pretreated for 1 h with the pan-caspase inhibitor Z-VAD-FMK (LKT Laboratories, Z8401), the IP_3_ receptor inhibitor 2-APB (FUJIFILM Wako, 013–24911), or the MCU inhibitor ruthenium red (FUJIFILM Wako, 189–03701), prior to the addition of test compounds.

#### *In vivo* luciferase imaging analysis

*In vivo* bioluminescence imaging was performed using an *in vivo* imaging system (IVIS; PerkinElmer). Prior to imaging, the fur at the measurement site was shaved, and the mice were anesthetized with isoflurane. D-Luciferin (Promega, P1043) was administered intraperitoneally at a dose of 150 mg/kg body weight in accordance with the manufacturer’s instructions. Bioluminescence signals were acquired starting 5 min after luciferin injection and quantified using Living Image software (Version 4.5.5, PerkinElmer). Luciferase activity was measured to assess the *in vivo* presence and dynamics of luciferase-expressing cells.

#### Matrigel transplantation

Senescent (IR-treated) or non-senescent mouse ear fibroblasts derived from p16^tTA−Luc^ mice were mixed with Matrigel (Corning, 35237) at a concentration of 2.5 × 10^6^ cells/mL. A total of 0.5 × 10^6^ cells (0.2 mL of Matrigel mixture) were injected subcutaneously into the dorsal flank of 12-week-old C57BL/6 mice using a 23-gauge syringe. Three days post-transplantation, luminescence was measured using IVIS to assess luciferase activity. After baseline imaging, the mice were provided with 0.05% Rosea-containing drinking water *ad libitum*, and a second IVIS measurement was performed 7 days after treatment initiation.

#### Skin aging model

To establish a skin aging model,^34^ 8-week-old male p16^tTA−Luc^ mice were subjected to ionizing radiation (IR). The dorsal hair was trimmed using an electric shaver, followed by baseline IVIS imaging. The exposed skin was then irradiated with 14 Gy of X-rays. Fourteen days post-irradiation, the dorsal area was re-shaved, and IVIS imaging was repeated to assess senescence induction. Thereafter, a 0.05% Rosea-containing solution was administered via drinking water, and IVIS imaging was performed again 4 weeks after the initiation of Rosea treatment.

#### RNA analysis

Total RNA was extracted from tissue and cell samples using either QIAzol Lysis Reagent (QIAGEN, 79306) or the Maxwell RSC 48 Instrument (Promega) in combination with the Maxwell RSC simplyRNA Tissue Kit (Promega, AS1340), according to the manufacturers’ protocols. Complementary DNA (cDNA) was synthesized, and real-time quantitative PCR (qPCR) was performed using SYBR Green Master Mix (Applied Biosystems, A25742) on a QuantStudio 6 Pro Real-Time PCR System (Applied Biosystems), following the manufacturer’s instructions. Primer sequences used for amplification are listed below. Housekeeping genes (*RPLP0*, *GAPDH*, *Rplp0*, or *Gapdh*) were used as internal controls for normalization.

Human primers (forward, backward):

*RPLP0*: 5′-TCTACAACCC TGAAGTGCTTGAT-3′, 5′-CAATCTGCAGACAGACACTGG-3′

*GAPDH*: 5′-CCCCGGTTTCTATAAATTGAGC-3′, 5′-CACCTTCCCCATGGTGTCT-3′

*CDKN1A*: 5′-CGAAGTCAG TTCCTTGTGGAG-3′, 5′-CATGGGTTCTGACGGACAT-3′

*CDKN2A*: 5′-GTGGACCTGGCTGAGGAG-3′, 5′-CTTTCAATCGGGGATGTCTG-3′

Mouse primers (forward, backward):

*Rplp0*: 5′-GATGCCCATGGGAAGACAG-3′, 5′-ACAATGAAGCATTTTGGATAA-3′

*Gapdh*: 5′-AGCTTGTCATCAACGGGAAG-3′, 5′-TTTGATGT TAGTGGGGTC TCG-3′

*Cdkn1a*: 5′-TCCACAGCGATATCCAGACA-3′, 5′-GGACATCACCAGGATTGGAC-3′

*Cdkn2a*: 5′-CGACGGGCATAGCTTCAG-3′, 5′-GCTCTGCTCTTGGGATTGG-3′

*Gpnmb*: 5′-ACGGCAGGTGGAAGGACT-3′, 5′-CGGTGAGTCACTGGTCAGG-3′

*Ccl2*: 5′- CATCCACGTGTTGGCTCA -3′, 5′- GATCATCTTGCTGGTGAATGAGT -3′

*Mmp12*: 5′- TTGTGGATAAACACTACTGGAGGT -3′, 5′- AAATCAGCTTGGGGTAAGCA -3′

*Cxcl*1: 5′- AGACTCCAGCCACACTCCAA -3′, 5′- TGACAGCGCAGCTCATTG -3′

*Il-1beta*: 5′- TGTAATGAAAGACGGCACAC -3′, 5′- TCTTCTTTGGGTATTGCTTGG -3′

#### Western blot analysis

Protein lysates were prepared using radioimmunoprecipitation assay buffer (10 mM Tris-HCl, pH 8.0; 140 mM NaCl; 5 mM EDTA; 0.025% NaN_3_; 1% Triton X-100; 1% deoxycholate; and 0.1% SDS) supplemented with a protease inhibitor cocktail (Roche, 11873580001). Equal amounts of protein were separated by SDS-polyacrylamide gel electrophoresis (SDS-PAGE) and transferred onto polyvinylidene difluoride (PVDF) membranes. Membranes were blocked with 5% skim milk in Tris-buffered saline with Tween 20 and incubated overnight at 4°C with primary antibodies against p53 (Leica, NCL-L-p53-CM5p) or α-tubulin (Cell Signaling, 2144), each diluted 1:1000. After washing, membranes were incubated with horseradish peroxidase (HRP)-conjugated anti-rabbit IgG secondary antibody (Jackson ImmunoResearch, 115-035-144) at a dilution of 1:5000. Immunoreactive bands were visualized using the Amersham ECL Prime detection reagent (Cytiva, RPN2232), and images were acquired using either the Amersham Imager 680 (Cytiva) or Fusion Solo S imaging system (Vilber).

#### FACS analysis

Cell death was assessed by flow cytometry using Propidium Iodide (PI) staining. HUVECs were treated with 20 μg/mL of Rhodiola rosea extract (Rosea) for 48 h and subsequently detached from the culture dish using trypsin. The cells were then stained with PI (5 μg/mL, BD Biosciences, 51-66121E), following the manufacturers’ protocols. Stained cells were analyzed using the Spectral Cell Analyzer ID7000 (Sony), and the proportions of PI-positive cells were quantified.

#### Systemic metabolic parameter

For the glucose tolerance test (GTT), mice were fasted for 6 h and subsequently administered glucose via intraperitoneal injection at a dose of 1 g/kg body weight in the early afternoon. For the insulin tolerance test (ITT), mice received an intraperitoneal injection of human insulin at a dose of 1 U/kg body weight. Blood samples were collected from the tail vein at 0, 15, 30, 60, and 120 min after injection, and blood glucose concentrations were measured using a glucose analyzer (Sanwa Kagaku Kenkyusho, Japan).

#### Physiological analyses

Motor coordination and balance were assessed using the rotarod test (O’Hara & Co., RRAC-3002). Mice were pre-trained for 2 min at speeds of 4, 6, and 8 rpm over a period of 3 consecutive days. On the test day, the rotation speed was gradually increased from 4 to 40 rpm over 300 s, and the latency to fall was recorded. Each mouse underwent three trials, and the average latency was calculated.

Endurance capacity was evaluated using a treadmill running test. Mice were trained over 3 days to run on a 5° incline at speeds of 5, 7, and 9 m/min for 2 min each. On the test day, the mice ran on the same incline starting at 5 m/min, with the speed increasing by 2 m/min every 2 min. The total running distance until exhaustion was recorded.

Spontaneous activity levels were monitored using a running wheel system (Muromachi Kikai, MK-713). Mice were housed individually in cages equipped with a running wheel for 3 days, with the first day allotted for acclimation and the subsequent 2 days allocated for measurement. The number of wheel rotations was recorded hourly, and the mean number of rotations on days 2 and 3 was calculated.

#### Quantification of hair loss and gray hair areas

In the aged mouse study, the extent of hair loss and gray hair was quantified using Fiji software (ImageJ version 1.53q).^35^ The affected area was calculated by dividing the total area of hair loss and gray hair by the overall skin surface area.

#### Histological analyses

Mouse tissues were fixed in 10% paraformaldehyde immediately after collection. The samples were subsequently embedded in paraffin, and serial 3 μm sections were prepared. Hematoxylin and eosin (H&E) staining and Masson’s trichrome staining were performed according to standard protocols. The stained sections were visualized using a BZ-9000 microscope (Keyence Corporation).

For immunofluorescence staining, paraffin sections were deparaffinized and subjected to antigen retrieval by microwave treatment in 10 mM Tris-EDTA buffer (pH 9.0) for 10 min. After blocking with bovine serum albumin, sections were incubated overnight with LaminB1 (Abcam, ab133741) and WGA488 (ThermoFisher SCIENTIFIC, W11261) at a dilution of 1:100. This was followed by a 1-h incubation with Goat Anti-Rabbit IgG H&L Alexa Fluor 647 (Abcam, ab150079) at 1:100 and Hoechst 33258 (Invitrogen, H3569) at 1:1000. Samples were imaged using an ECLIPSE Ti2 confocal microscope (Nikon Instruments Inc.).

Time-lapse phase-contrast images of cellular morphology were acquired using the Incucyte SX5 (Sartorius AG).

For ultrastructural analysis, cells were fixed with 2.5% glutaraldehyde and processed for transmission electron microscopy. Electron micrographs were acquired using a JEM1400 transmission electron microscope (JEOL).

All histological and imaging procedures, including tissue processing, sectioning, electron microscopy, and staining, were performed with the technical assistance of the Laboratory of Morphology and Image Analysis, Biomedical Research Core Facilities, Juntendo University Graduate School of Medicine.

#### SA-β-gal staining

Cells were fixed with 0.2% glutaraldehyde and incubated at 37°C for 4 h in a senescence-associated β-galactosidase (SA-β-gal) staining solution containing 1 mg/mL X-gal (Takara, 9031), 5 mM potassium ferricyanide, 5 mM potassium ferrocyanide, 150 mM NaCl, 2 mM MgCl_2_, 0.01% sodium deoxycholate, and 0.02% Nonidet P-40, adjusted to pH 6.0. After staining, the cells were washed with PBS, and SA-β-gal–positive cells were quantified by microscopic examination. The percentage of SA-β-gal-positive cells was calculated as the SA-β-gal positivity rate.

For tissue staining, gonadal white adipose tissue (gWAT) was harvested from mice, rinsed with PBS, and incubated in the same SA-β-gal staining solution at 37°C for 3 h. The tissue was subsequently fixed in 4% paraformaldehyde, and the staining intensity of SA-β-gal activity was quantified using Fiji software (ImageJ).

#### Imaging of ER and mitochondria

Human umbilical vein endothelial cells (HUVECs) were seeded onto glass-bottom dishes and treated with EGC-EGCG (12.5 μM) for 24 h. Cells were subsequently incubated with 0.4 μM MitoTracker Green FM (Thermo Fisher Scientific, M7514) and 1 μM ER-Tracker Red (Thermo Fisher Scientific, E34250), both diluted in Hank’s Balanced Salt Solution (HBSS), for 30 min at 37°C. After washing with HBSS, live-cell imaging was performed using a confocal laser scanning microscope.

#### Measurement of intracellular Ca^2+^ levels

To assess mitochondrial calcium levels, cells were incubated for 30 min at 37°C with 2.5 μM Rhod-2 AM (Thermo Fisher Scientific, R1245MP) and 0.4 μM MitoTracker Green FM under standard culture conditions. After staining, cells were washed with PBS and treated with EGC-EGCG (12.5 μM) for 1 h at 37°C in 5% CO_2_. Confocal microscopy was used to visualize intracellular calcium, and Rhod-2 AM fluorescence was normalized to MitoTracker Green signals to quantify mitochondrial calcium levels.

For flow cytometry analysis, cells were treated with EGC-EGCG (12.5 μM) for 24 h, washed with PBS, and then incubated with 2.5 μM Rhod-2 AM for 30 min. Following staining, cells were detached using trypsin and analyzed by flow cytometry.

### Quantification and statistical analysis

#### Statistics and reproducibility

All statistical analyses were performed using SPSS version 28.0.1.0 (IBM), and graphs were generated using RStudio (R version 4.4.0). Statistical details for each experiment can be found in the figure legends, as indicated, including the statistical test used, the exact value of n. All data were derived from independent biological replicates. Data presentation included combined dot plots (showing individual data points) and box-and-whisker plots. In these plots, the boxes represent the interquartile range (25th to 75th percentiles), with the median indicated by a solid line and whiskers extending to the minimum and maximum values. In some cases, data were expressed as mean ±2 × standard error of the mean (SEM). Outliers and extreme values were not excluded from the analysis.

Statistical comparisons between 2 groups were conducted using two-tailed Student’s t-tests. For comparisons involving three or more groups, one-way or two-way analysis of variance (ANOVA) was performed, followed by Tukey’s post hoc test for multiple comparisons. Repeated measures ANOVA was used for time-course data analysis. A *p* value of less than 0.05 was considered statistically significant. Asterisks indicate statistical significance as follows: ∗*p* < 0.05, ∗∗*p* < 0.01.

Sample sizes were based on previous experimental data; no statistical methods were used to predetermine sample sizes for the experiments referenced here.^12^^,^^13^^,^^36^ Mice were allocated to experimental groups to ensure similar baseline characteristics such as body weight and physical activity levels. Investigators were blinded to group allocation during IVIS imaging, systemic metabolic assessments, and physiological analyses. However, blinding was lifted for downstream data analysis following sample collection. Blinding was not applied to *in vitro* experiments.
