# Supplementary material for: Natural senolytic activity of Rhodiola rosea extract alleviates age-associated phenotypes via paraptosis
Source: iScience. 2026 Apr 4;29(5):115607. doi: 10.1016/j.isci.2026.115607 (PMC13101718; doi:10.1016/j.isci.2026.115607)
Supplement: Document S1. Figures S1–S4, and Data S1 and S2 [file mmc1.pdf]

iScience, Volume *29*

Supplemental information

Natural senolytic activity of *Rhodiola rosea* extract alleviates age-associated phenotypes via paraptosis

Ryo Furuuchi, Yohko Yoshida, Goro Katsuumi, Takaaki Furihata, Yusuke Joki, Chieh-Lun Hsiao, Masayoshi Suda, Hana Saito, Tamano Kumazaki, Hidefumi Makabe, Manabu Abe, Ippei Shimizu, and Tohru Minamino

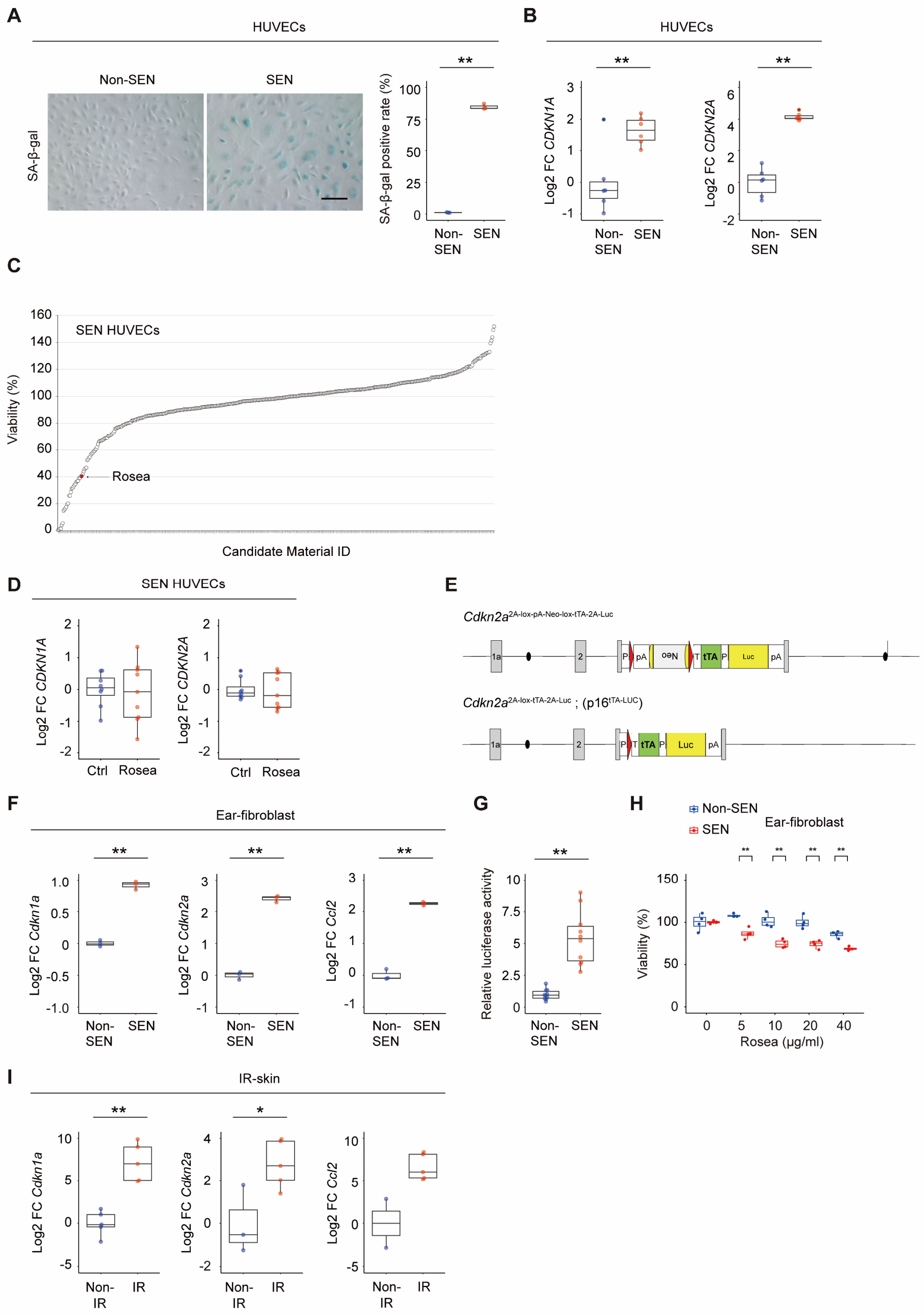

Supplementary Figure 1 Confirmation of cellular senescence and senolytic effects of Rosea.

(A) Representative images of senescence-associated β-galactosidase (SA-β-gal) staining and quantification of SA-β-gal-positive cells in non-senescent (Non-SEN) and replicative

senescent (SEN) human umbilical vein endothelial cells (HUVECs) (n = 3 each). Scale bar, 100 µm. **(B)** Relative mRNA expression levels of *CDKN1A* and *CDKN2A* in Non-SEN and SEN HUVECs (n = 6 each). **(C)** Primary screening of plant-derived extracts in SEN HUVECs. SEN HUVECs were treated with 481 plant-derived extracts at 12.5 µg/mL, and cell viability was measured to identify extracts that reduce the viability of senescent cells. **(D)** SEN-HUVECs were treated with Rosea (10 µg/mL) for 24 h, and the mRNA expression levels of *CDKN1A* and *CDKN2A* (n = 9 each). **(E)** Schematic of the *Cdkn2a* ($p16^{Ink4a}$) knock-in reporter allele in which a 2A-lox-pA-Neo-lox-tTA-2A-Luc cassette was inserted into the *Cdkn2a* locus (top). Upon Cre recombination, the lox-flanked STOP/Neo cassette (pA-Neo) is excised, generating the Cre-excised allele ($p16^{tTA\text{-}Luc}$; $Cdkn2a^{2A\text{-}lox\text{-}tTA\text{-}2A\text{-}Luc}$) in which tTA and luciferase are expressed under the control of the endogenous *Cdkn2a* ($p16^{Ink4a}$) promoter (bottom). **(F)** Relative mRNA expression levels of *Cdkn1a*, *Cdkn2a*, and *Ccl2* in non-senescent and senescent ear-derived fibroblasts isolated from $p16^{tTA\text{-}Luc}$ mice, following senescence induction by 10 Gy X-ray irradiation (IR) (n = 3 each). **(G)** Relative luciferase activity of non-senescent and senescent mouse ear fibroblasts (n=10 each). **(H)** Viability assays of Non-SEN and IR-induced SEN mouse ear fibroblasts treated with *Rhodiola rosea* extract (Rosea) for 48 hours (n = 4 each). **(I)** Relative mRNA expression levels of *Cdkn1a*, *Cdkn2a*, and *Ccl2* in IR-exposed versus non-IR skin tissues from $p16^{tTA\text{-}Luc}$ mice (n = 5 each). *Ccl2* expression in non-IR skin was undetectable in 3 out of 5 samples, and thus statistical testing was not performed for this gene. Statistical analysis was conducted using a two-tailed Student's *t*-test. Sample sizes (n) indicate independent biological replicates for in vitro experiments and mice for in vivo experiments. $P^* < 0.05$; **P****< 0.01. All data are presented as box-and-whisker plots showing the data range (whiskers), interquartile range (box), median (solid line), and individual values (dots).

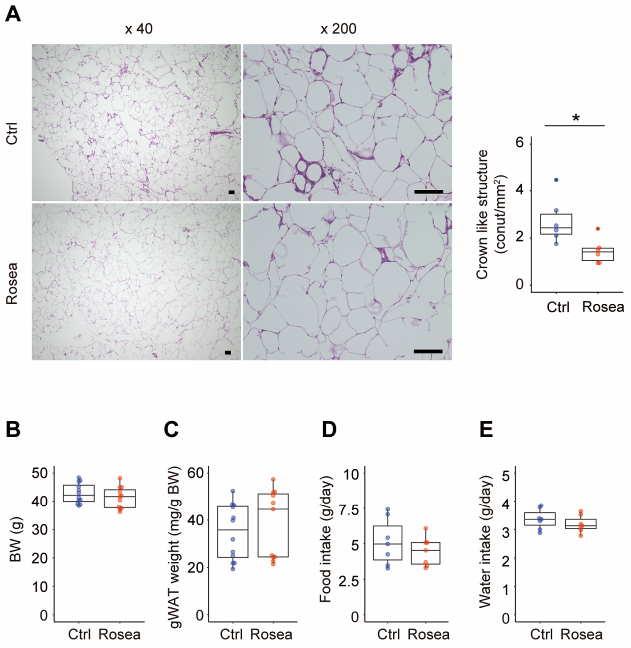

Supplementary Figure 2 Effect of Rosea administration on body weight and food/water intake in obese mice.

(A) Representative images and quantitative analysis of crown-like structures in gonadal white adipose tissue (gWAT) from high-fat diet (HFD)-induced obese mice treated with control or Rosea for 4 weeks. Scale bar, 100 µm. (n = 6 each). **(B)** Body weight (BW) of obese mice (n = 12 control, 11 Rosea). **(C)** gWAT weight normalized to body weight in obese mice (n = 12 control, 11 Rosea). **(D)** Daily food intake averaged over 3 consecutive days in obese mice (n = 7 per group). **(E)** Daily water intake measured over the same period (n = 7 per group). Data are presented as box-and-whisker plots, where boxes indicate the interquartile range (25th-75th percentiles), whiskers represent the full data range, the solid line indicates the median, and individual data points are shown as dots. Sample sizes (n) indicate mice per group. P* < 0.05. Statistical significance was assessed using a two-tailed Student’s t-test.

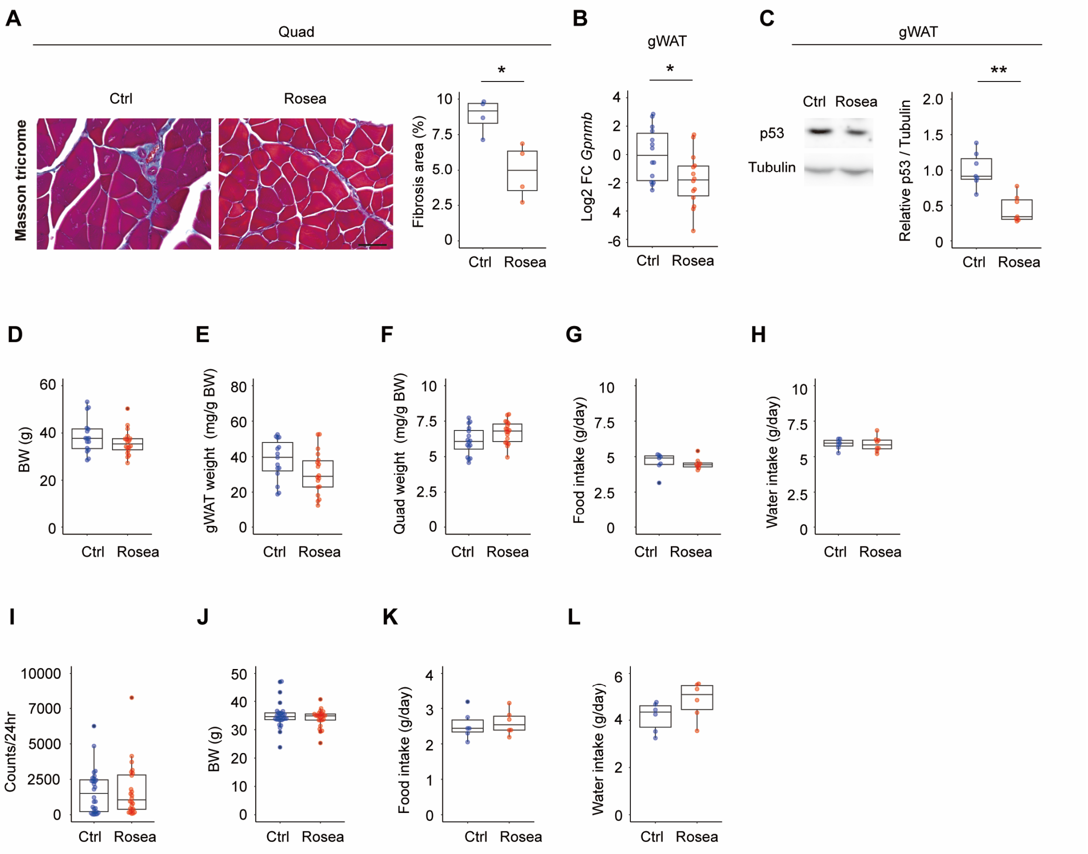

Supplementary Figure 3 Effects of Rosea treatment on physiological and molecular parameters in middle-aged and aged mice.

(A) Quantification of fibrotic area in quadriceps muscle (Quad) sections from middle-aged mice stained with Masson's trichrome (n = 4 per group). Scale bar, 50 µm. **(B)** Relative mRNA expression levels of *Gpnmb* in gWAT from middle-aged mice (n = 14 per group). **(C)** Relative expression levels of p53 protein in gonadal white adipose tissue (gWAT) from middle-aged mice treated with control or Rosea (n = 7 per group). The unmodified blot image corresponding to Figure S3C is provided in Data S2. **(D)** Body weight (BW) of middle-aged mice treated with control or Rosea (n = 16 control, 18 Rosea). **(E)** gWAT weight normalized to BW in middle-aged mice (n = 15 control, 18 Rosea). **(F)** Quad weight normalized to BW in middle-aged mice (n = 16 control, 18 Rosea). **(G)** Daily food intake in middle-aged mice averaged over 3 days (n = 7 control, 8 Rosea). **(H)** Daily water intake in middle-aged mice (n = 7 control, 8 Rosea). **(I)** Daily activity levels of aged mice measured using a wheel running apparatus, expressed as average daily counts (n = 28 control, 24 Rosea). **(J)** BW of aged mice after Rosea administration (n = 28 control, 24 Rosea). **(K)** Daily food intake of aged mice (n = 6 per group). **(L)** Daily water intake of aged mice (n = 6 per group). Data are shown as box-and-whisker plots: boxes indicate the interquartile range (25th-75th percentiles), whiskers represent the minimum and maximum values, the solid line indicates the median, and individual data points are shown as dots. Statistical significance was determined using a

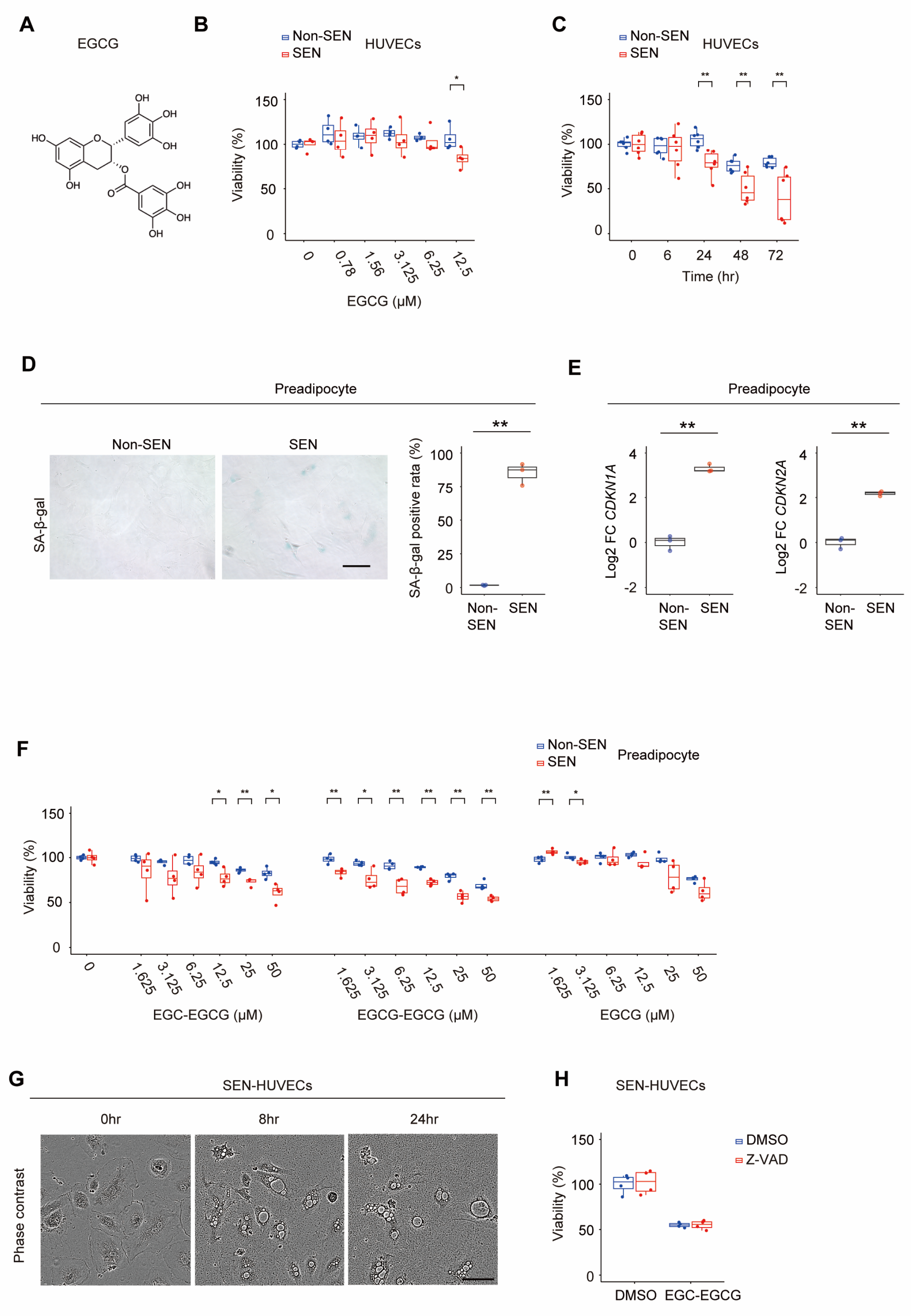

two-tailed Student's t-test. Sample sizes (n) indicate mice per group. *P < 0.05, **P < 0.01 were considered statistically significant.

Supplementary Figure 4 Senolytic activity of Rhodiola-derived active components in various senescent cell models.

(A) Chemical structure of epigallocatechin gallate (EGCG). **(B)** Viability of non-senescent (Non-SEN) and replicative senescent (SEN) HUVECs after treatment with

EGCG for 48 hours, assessed by a cell viability assay (n = 4 per group). **(C)** Time-course analysis of viability in Non-SEN and SEN HUVECs treated with EGC-EGCG at various time points (n = 6 per group). **(D)** SA-β-galactosidase staining images and quantification of SA-β-gal-positive cells in Non-SEN and SEN human preadipocytes (n = 3 per group). **(E)** Relative mRNA expression levels of senescence-associated genes *CDKN1A* and *CDKN2A* in Non-SEN and SEN preadipocytes (n = 3 per group). **(F)** Viability of Non-SEN and SEN preadipocytes treated for 48 hours with EGC-EGCG, EGCG-EGCG, or EGCGG, evaluated using cell viability assays (n = 4 per group). **(G)** Time-lapse phase-contrast images of SEN HUVECs treated with Rosea (20 μg/mL) showing time-dependent morphological changes. Scale bar, 100 μm. **(H)** Viability of SEN HUVECs treated with 12.5 μM EGC-EGCG or DMSO for 48 hours following pretreatment with the pan-caspase inhibitor Z-VAD-FMK (10 μM) to evaluate caspase-dependent cell death (n = 4 per group). Data are presented as box-and-whisker plots: boxes represent the interquartile range (25th-75th percentiles), whiskers denote the full data range, the solid line indicates the median, and individual data points are plotted as dots. Statistical significance was assessed using a two-tailed Student's t-test (**B–F**) or two-way ANOVA followed by Tukey's multiple comparisons test (**H**). Sample sizes (n) indicate independent biological replicates per group. *$P < 0.05$, **$P < 0.01$ were considered statistically significant.

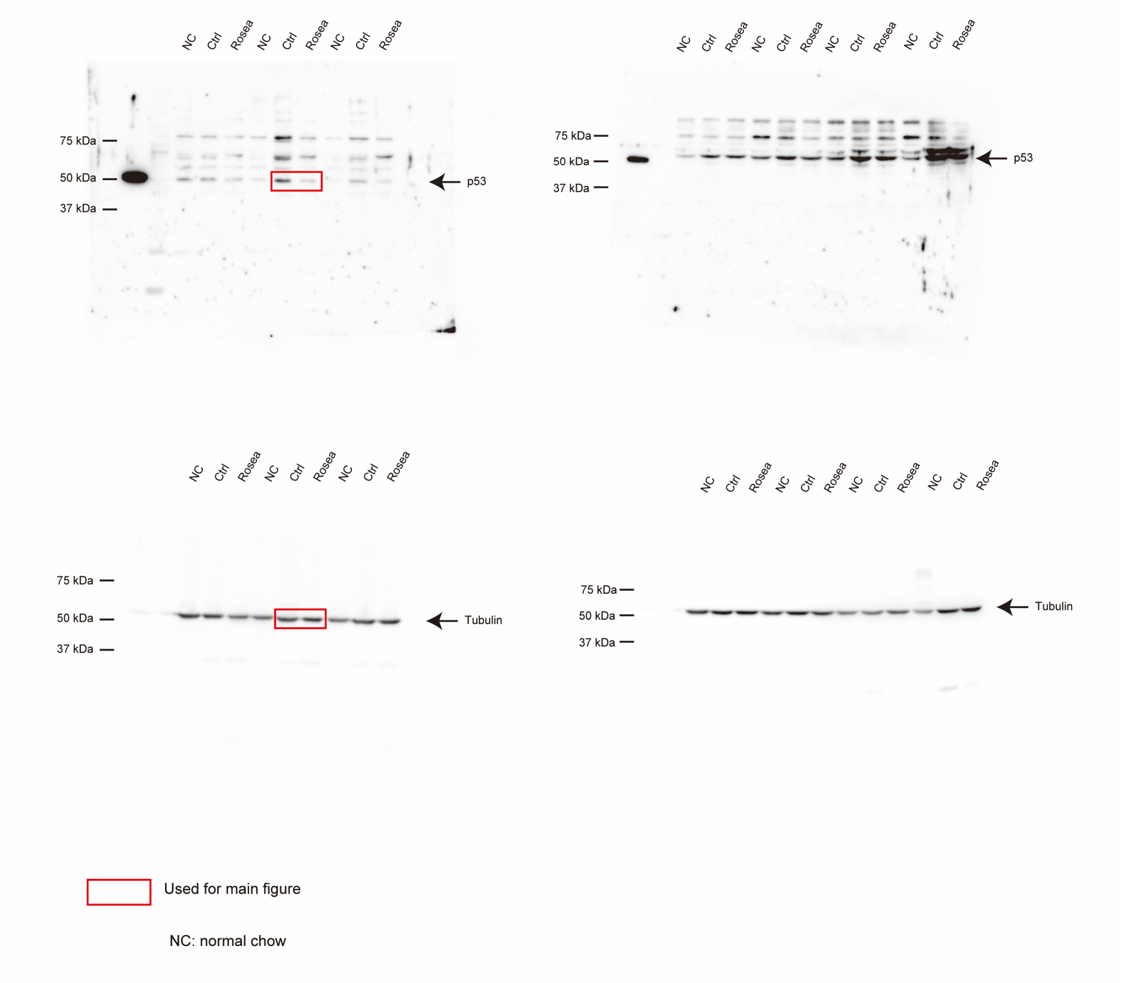

Data S1 Unmodified blot images corresponding to Figures 2E.

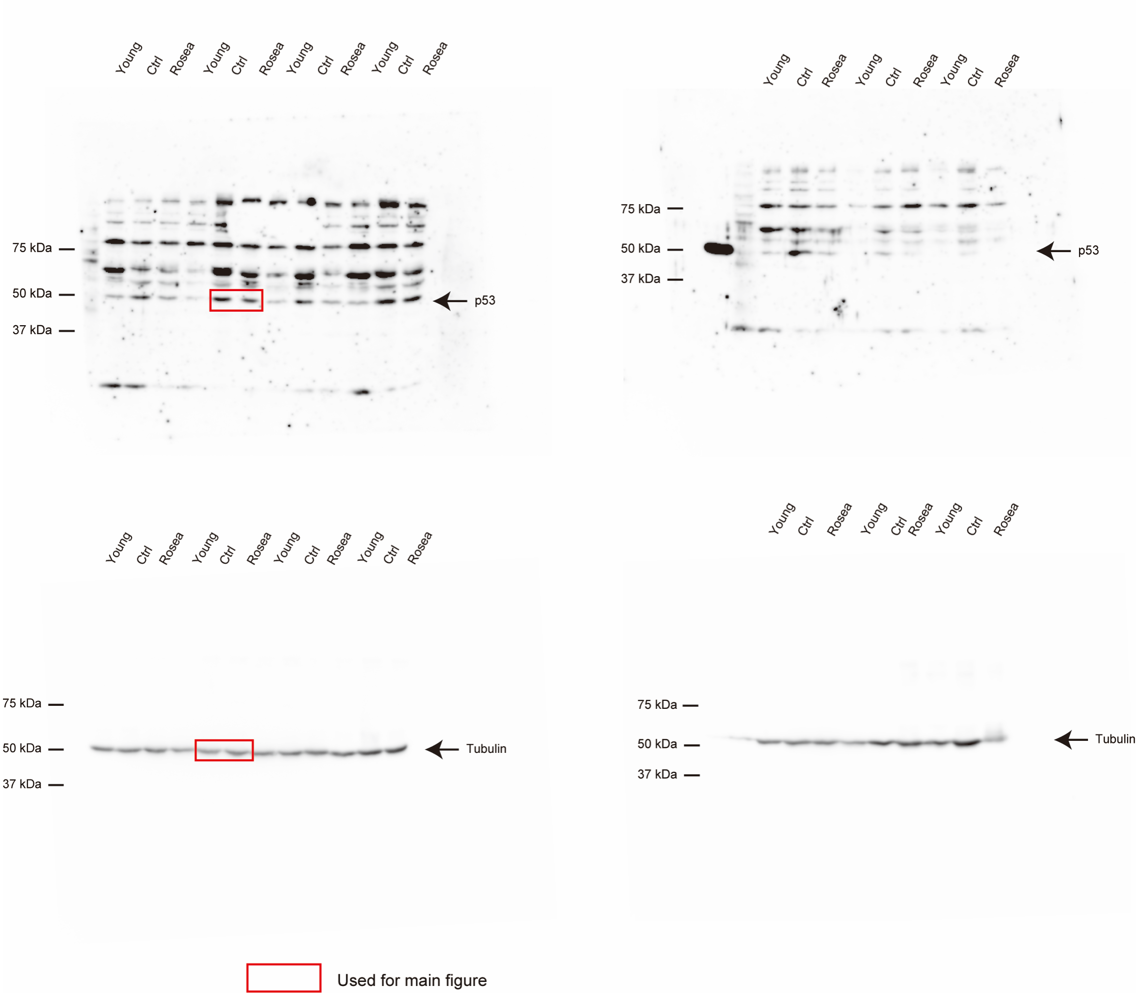

Data S2 Unmodified blot images corresponding to Figures S3C.
